# Supplementary material for: Generating intense electric fields in 2D materials by dual ionic gating
Source: Nat Commun. 2022 Nov 3;13:6601. doi: 10.1038/s41467-022-34158-z (PMC9633598; doi:10.1038/s41467-022-34158-z)
Supplement: Supplementary file 1 — Supplementary Information [file 41467_2022_34158_MOESM1_ESM.pdf]

Supplementary information for

"Generating intense electric fields in 2D materials by dual ionic gating"

Benjamin I. Weintrub[1], Yu-Ling Hsieh[1,2], Sviatoslav Kovalchuk[1], Jan N. Kirchhof[1], Kyrylo Greben[1], and Kirill I. Bolotin*[1]

1. Department of Physics, Freie Universität Berlin, Berlin, Germany
2. Department of Mechanical Engineering, National Central University, Taoyuan City, Taiwan

Supplementary Note 1: Extracting gating efficiency, bandgap, and electric field

Our 2DM is exposed to two individual ILs, one above and one below the 2DM. Only a fraction of the potential applied to the gate electrodes (V_b, V_t) inside these liquids falls across the 2DM/IL interface (V_b^{ref}, V_t^{ref}), which is characterized by gating efficiencies: $\alpha_b \equiv V_b^{ref}/V_b$ and $\alpha_t \equiv V_t^{ref}/V_t$. These gating efficiencies depend mainly on the size of the gate electrodes (relative to the 2DM and exposed drain/source electrodes). In our transport devices, we try to equalize these parameters between top and bottom gate electrodes. The size, material composition, and placement of the gate electrodes above and below the 2DM are roughly equal. Because of this symmetry between top and bottom gate electrodes, we assume $\alpha_b = \alpha_t = \alpha$. Similarly, we can relate (V_g, ΔV) to the fraction of those applied voltages, (V_g^{ref}, ΔV^{ref}), and when we assume $\alpha_b = \alpha_t = \alpha$, we see that $\alpha = V_g^{ref}/V_g = \Delta V^{ref}/\Delta V$. Therefore, everywhere in the main text, we just use a single parameter, α, without specifying which gate it refers to.

We can extract α directly from reference voltage measurements (ΔV^{ref} vs. ΔV data in Fig. 5a). We form a linear fit and note that the slope is $\Delta V^{ref}/\Delta V = \alpha \approx 90$ % for sample #4 in Fig. 5a (red curve). We also see in Supplementary Figure 2 that the voltage drops across both gate EDLs as a function of ΔV are almost the same, confirming that $\alpha_b \approx \alpha_t$ in this device. In samples of Fig. 3 and 4, we do not measure references voltages simultaneously, so instead we extract α from the transport data (see below).

We model our devices, which include effects from free carrier screening, based on existing models in the literature[1]. For a bilayer, we write:

$$e\alpha V_b = E_b + e\sigma_b/C \quad (1)$$
$$e\alpha V_t = E_t + e\sigma_t/C \quad (2)$$
$$\sigma_b = en_b + C_{int}d_{int}F_{int} \quad (3)$$
$$\sigma_t = en_t - C_{int}d_{int}F_{int} \quad (4)$$
$$F_{int} = (ed_{int})^{-1}(E_b - E_t) \quad (5)$$

Here, E_b and E_t are the Fermi level relative to the middle of the bandgap for bottom and top layers respectively, σ_b and σ_t are the charge densities of the bottom and top ionic liquids respectively gating the device, n_b and n_t are the carrier densities of the bottom and top layers respectively (positive for electrons, negative for holes), C and $C_{int} = \varepsilon_0\varepsilon_{2DM}/d_{int}$ are the areal IL/TMDC and interlayer TMDC capacitance respectively, d_{int} is the interlayer separation between adjacent layers in the TMDC, ε_{2DM} is the dielectric constant of the TMDC, and F_{int} is the electric field between bottom and top TMDC layers. Since we define the EDL distance to be from the ions to the outer surface of the 2DM, and Equations 1 and 2 above describe the chemical potential difference between ions and the center of the 2DM, the areal IL/TMDC

capacitance, C, must include the EDL and half of a monolayer. This means that $C = (1/C_{EDL} + 1/C_{½L})^{-1} = \varepsilon_0\varepsilon_{EDL}\varepsilon_{2DM}/(\varepsilon_{2DM}d_{EDL} + ½\varepsilon_{EDL}d_{int})$ where ε_{EDL} is the dielectric constant of the ionic liquid. Equations 1 and 2 are obtained by finding the total chemical potential difference between the ionic layers and the TMDC layer closest to it, Equations 3 and 4 are from Gauss's law, and Equation 5 is from chemical potential equilibrium between bottom and top layers of the bilayer. We numerically solve Equations 1 through 5 and plot maps of the field and layer-dependent carrier density (Supplementary Figure 4). Our simulations also include a moderate amount of localized bandgap states, 10^{12} cm^{-2}, and we see that in this case, the field and carrier density are practically unaffected, and the bandgap still closes at the same value, ~3 V/nm. In our bilayer simulations, we use E_{2L}^0 = 1.8 eV, α = 77.3 %, ε_{2DM} = 7.5, d_{int} = 0.6 nm, C = 12.5 μF/cm^2, and the typical energy-independent 2D density of states. Defects were simulated by assuming a continuous bandgap density of states such that when the Fermi level traverses the entire bandgap, a defect-related carrier density of 10^{12} cm^{-2} is generated in the material.

While for non-zero carrier density the results of Equations 1 – 5 are complicated, when the carrier density is zero, simple analytical expressions can be obtained. When the bilayer is considered as a compound material, its bandgap (E_{2L}) has interlayer character with the valence band localized in the top layer and conduction band in the bottom layer. Equations 1 – 5 can be used to find the (interlayer) bandgap from transport measurements. We define electron (hole) threshold voltages in the V_g-domain, V_e (V_h), correspond to the Fermi level at the conduction band minimum of the bottom layer (at the valence band maximum of the top layer). Assuming $n_b = n_t = 0$ (when Fermi levels of both layers are inside their respective bandgaps), we get $½e\alpha(V_e - V_h) = E_{2L}^0 - ed_{int}F_\perp = E_{2L}$. The system of equations can be generalized to N-layered TMDCs using the same approach of using Gauss's law and chemical potential differences, and a modified equation is obtained: $E_N = E_N^0 - e(N - 1)d_{int}F_\perp = ½e\alpha(V_e - V_h)$. In this case, $(N - 1)d_{int}$, takes the role of d_{int}. We see that the bandgap obtained from the transport data can be used to extract field strength.

Experimentally, we extract V_e and V_h from transport measurements by fitting a line to the electron and hole linear conduction regions of the I_{ds} vs. V_g data, and then we determine the value of V_g where the fit goes to zero. We extract threshold voltages like this at several values of ΔV. We form a linear fit to the $V_e - V_h$ vs. ΔV data, and this linear fit can be used to determine both α and the point at which the bandgap closes, ΔV_{close}. To calculate α, we use the value of $V_e - V_h$ when $\Delta V = 0$ V from the fit, and note that the bandgap in this case is equal to the zero-field bandgap, E_N^0, which leads to $\alpha = 2E_N^0 e^{-1}(V_e - V_h)^{-1}$, which we use to calculate the bandgap at non-zero ΔV, $E_N = ½e\alpha(V_e - V_h)$. Finally, we determine $F_\perp = (e(N - 1)d_{int})^{-1}(E_N^0 - E_N)$, which we use in the main text to analyze transport data.

Equations 1 – 5 show that $F_\perp$ can be related to the difference of reference voltages of top and bottom ionic liquids ($\Delta V^{ref} = \alpha V_b - \alpha V_t$). When the Fermi level is inside the bandgap (zero carrier density), we obtain $F_\perp = \Delta V^{ref}/d_\perp$, where $\Delta V^{ref} = \alpha\Delta V$ is the potential difference between bottom and top ionic liquids and $d_\perp = Nd_{int} + 2(\varepsilon_{2DM}/\varepsilon_{EDL})d_{EDL}$ is the effective distance between the ionic liquids. This equation is used to extract electric field strength from reference voltage measurements.

We can combine these equations for $E_N(F_\perp)$ with $F_\perp(\Delta V)$ to arrive at $E_N = E_N^0 - e(N-1)d_{int}F_\perp = E_N^0 - e\alpha\Delta V(N-1)d_{int}/(Nd_{int} + 2(\varepsilon_{2DM}/\varepsilon_{EDL})d_{EDL})$. This means that bandgap is related to ΔV through $(N-1)d_{int}/(Nd_{int} + 2(\varepsilon_{2DM}/\varepsilon_{EDL})d_{EDL})$. If we make the approximation $2(\varepsilon_{2DM}/\varepsilon_{EDL}) \approx 1$ and $d_{EDL} \approx d_{int}$, we see that bandgap is related to ΔV through $(N-1)/(N+1)$, which approaches 1 when N goes to infinity. This is another way of saying that as the number of layers, N, of the 2DM increase, more of the applied ΔV is dropped across the material compared to the empty space between ions and the 2DM, yielding a stronger dependence of E_N to $F_\perp$. This enables us to close the bandgap of few-layer WSe_2 much more easily than e.g. bilayer WSe_2.

The bandgap of monolayer WSe_2 does not depend on perpendicular field. In a control measurement, we fabricated a test device, sample #5, and tested this assumption (Supplementary Figure 3). As expected, we find no systematic dependence of bandgap on ΔV that is not caused by electrolyte instability.

Supplementary Note 2: Spurious or leakage current in our IL-gated devices

It is important to ensure that I_{ds} in our devices is separated from the leakage current across our 2DM/IL interface. Below, we show an approach to analyze the leakage current. We model our system as a combination of several capacitors (Supplementary Figure 5): C_b^w and C_t^w are working electrode capacitors corresponding to the interface between bottom and top gate electrodes and ILs respectively. The counter electrode capacitors, C_b^c and C_t^c, model the IL/2DM interface for bottom and top ILs respectively. Finally, $C_\perp$ models the direct coupling between bottom and top ILs, which couple through both the 2DM and the PMMA/SiN. We model leakage by introducing currents I_b^w, I_t^w, I_b^c, I_t^c, and $I_\perp$, each associated with their respective capacitor. These currents arise due to i) charging/discharging of the corresponding EDLs, or ii) charge transfer due to electrochemical reactions at the corresponding interface[2]. We note that when the 2DM is electrically floating, the 2DM acts as an ideal atomically thin barrier separating the two ILs. This barrier prevents electrochemical reactions between the two ILs while still enabling the study of the interaction between the two ILs when separated by an ultrathin charge-neutral membrane. Due to the expected suppression of electrochemical reactions in this state, we predict that our device geometry will provide a robust platform to study previously-inaccessible phenomena such as dielectric breakdown of the barrier material (without influence of damage from gate leakage currents) and interactions between ions at ultralow distances.

In this model, I_b^w and I_t^w are the currents experimentally detected by our sourcemeters applying the corresponding gate voltages. The currents I_b^c and I_t^c are the leakage currents flowing in the 2DM and potentially contributing to the measured drain-source current. By invoking Kirchhoff's current law for the circuit in Supplementary Figure 5, we get $I_b^w + I_t^w = I_b^c + I_t^c$. This means that we can determine the maximum amount of leakage current which could affect our measured I_{ds} by simply taking the sum of leakage currents detected by the sourcemeters. The value of $I_b^c + I_t^c$ in our devices is at most a few nA, at least one order of magnitude lower than the scale of I_{ds} used to obtain threshold voltages.

In our dual IL-gated FETs without any capping layer above the 2DM, current may flow through the suspended region of interest as well as supported regions of the flake, I_{susp} and I_{supp} respectively. We model this as a parallel connection of two different gate-tunable

resistors, one resistor modeling the suspended dual-gated region of the 2DM (Fermi level depends on $V_b + V_t$), and the other resistor models the SiN-supported region of the 2DM (Fermi level depends on just V_t). The total drain-source current is $I_{ds} = I_{susp} + I_{supp}$.

We performed simple test measurements in order to confirm the PMMA capping layer's ability to neutralize the contribution of I_{supp} (see Supplementary Figure 6). We fabricated a simple 2L WSe_2 device using the same fabrication methods outlined the Methods section, but on a different substrate (300 nm SiO_2 thermally grown on Si). The device has two channel regions, one without PMMA (Ch1) and one covered by crosslinked PMMA (Ch2). We apply a drop of DEME-TFSI on top of the WSe_2, and then we perform transport measurements of Ch1 and Ch2 simultaneously (see Supplementary Figure 6). It is clear from the data that crosslinked PMMA blocks ions from changing the carrier density in the covered region, Ch2, and given the intrinsic charge-neutrality of the WSe_2, we see that no current flows through the covered channel.

Supplementary Note 3: Detecting ruptured devices

It is crucial to ensure that the suspended 2D material is intact, because a rip or tear in the suspended region will bring the bottom and top ionic liquids into contact which will short them together and prevent generation of a perpendicular electric field. In reference voltage measurements where the top and bottom ionic liquid reservoirs are in contact with each other, we expect a near-zero difference between top and bottom ionic liquid potentials (reference voltages). To confirm this, we compared the reference voltage difference (ΔV^{ref}) vs. applied voltage difference (ΔV) for an intact sample (1L WSe_2, sample #4) against a bare substrate with an uncovered hole which causes top and bottom ionic liquids to touch through the hole in the SiN (Supplementary Figure 7).

As expected, we observe a much larger ΔV^{ref} for the intact device. In addition, we note orders-of-magnitude different leakage currents between ruptured and unruptured samples. The leakage current is from electrochemical reactions at the gate electrodes, which is an indicator of how much voltage is dropped at each gate. In an intact device (3-capacitor system in Supplementary Figure 2a), most of the applied ΔV drops across the intact 2DM, causing a small voltage drop at the top/bottom gate electrodes and hence a small leakage current. However, a ruptured device (2-capacitor system) shorts the two liquids together (meaning no voltage drop across the 2DM), and all of the applied ΔV drops across the gate electrodes, strongly driving electrochemical reactions at the gates and hence causing a large leakage current. Overall, electrochemical measurements allow us to quickly and effectively determine whether a sample is ruptured or not.

Supplementary Note 4: Field dependence of the OFF current

We can confirm field-dependent closing of the bandgap in our multilayer system via measurements of the "off" current of the TMDC transistor. When the Fermi level is inside the bandgap, E_{gap}, the current is given by $I_{OFF} \sim n_{intrinsic} \sim \exp(-E_{gap}/2k_BT)$ where I_{OFF} is the minimum current measured inside the bandgap and $n_{intrinsic}$ is the intrinsic carrier concentration of thermally excited carriers[3]. In conventional semiconductor physics, the variation of I_{OFF} vs. T can be used to determine E_{gap}. In our case, since the temperature cannot be changed, the

variation of I_{OFF} with perpendicular field at constant T can be used to confirm the field dependence of E_{gap}.

To carry out these I_{OFF} vs. E_{gap} measurements, we take line cuts of our I_{ds} vs. (V_b, V_t) transport map from our 4L device. We found I_{OFF} (minimum current inside the bandgap) at each ΔV. We then plot I_{OFF} vs. E_{gap}, where E_{gap} is solved for as a function of ΔV using methods from the main text (Fig. 3). As expected, I_{OFF} increases exponentially when the bandgap is decreased. Finally, we fit our data with $I_{OFF} = I_0\exp(-E_{gap}/2k_bT)$ with temperature T as a free parameter. From the fit, we obtain $T \approx 440 \pm 10$ K, close to the measurement temperature of ~300 K. We find this agreement as good, given that our data does include effects from contact resistance (which dominate I_{OFF} for small gaps), the contribution of in-gap states (which are large when the gap is almost closed), or leakage through e.g. electrolyte (which is small but can dominate the data for large bandgap). The results of this are summarized in Supplementary Figure 9.

Supplementary Note 5: Interlayer exciton photoluminescence

Optical measurements are one of the most direct approaches to measure bandgaps. However, the interlayer exciton in WSe_2 is indirect and has low oscillator strength[4]. Also, our ionic liquid luminesces weakly in the range of interlayer excitons, enough to dominate weakly-luminescing interlayer excitons. To overcome both problems, we perform photoluminescence (PL) measurements on a related system which is free from these issues. We fabricate a suspended $MoSe_2/WSe_2$ heterobilayer, where an interlayer exciton near 1.35 eV is the ground state of the system (Supplementary Figure 10a) with a large-enough oscillator strength to be clearly observed at room temperature. The interlayer exciton energy depends on the interlayer bandgap, which is tunable with a perpendicular electric field, and can therefore be used as a probe to measure the field. We also use NaCl dissolved in water as our electrolyte instead of our usual ionic liquid. This NaCl solution has a much lower capacitance of the ionic double layer, ~0.3 μF/cm^2 for a molarity of ~10 μM, compared to our DEME-TFSI ionic liquid, > 10 μF/cm^2, and the maximum field from the NaCl solution is correspondingly lower than that from the ionic liquid[5,6]. While less effective at producing fields than ionic liquids, this system is free from spurious photoluminescence and can be used with an immersion objective, thereby enabling high-resolution measurements. While the details of this system are of course different from that used in the main text, optical measurements of the $MoSe_2/WSe_2$ heterobilayer can be used to confirm the ability of our ionic gating approach to controllably generate fields.

To probe the field-dependent bandgap of this device, we examined the PL from our $MoSe_2/WSe_2$ heterostructure suspended in 10 mM NaCl dissolved in water vs. $\Delta V \equiv V_b - V_t$. To measure PL, we used a high numerical aperture objective immersed into the electrolyte. At zero ΔV, we observe a peak at ~1.335 eV. With increased ΔV up to 0.5 V, the peak blueshifts by $\Delta E \approx 15$ meV to ~1.350 eV. This is the expected result of increased voltage oriented from $MoSe_2$ to WSe_2 (Supplementary Figure 10b). The symmetry between the layers of the $MoSe_2/WSe_2$ heterobilayer is broken even at zero field, unlike in homobilayer WSe_2. The application of a field in one direction redshifts the exciton, and a field in the other direction blueshifts the interlayer exciton[7]. As the oscillator strength is higher for the blueshifted

interlayer exciton, it is easier for us to observe it. We estimate the field inside the heterostructure from bandgap modification as $F_{□} = \Delta E/ed_{int}$ = 25 mV/nm, where $d_{int} \approx 0.6$ nm is the interlayer distance.

We can also compare this field extracted from PL measurements to the expected field inside the ionic double layer, which we estimate using the same techniques as in the manuscript. We recall that $F_{□} = \Delta V^{ref}/d_{□} \approx \Delta V^{ref}/(2d_{Debye})$, where the reference voltage, ΔV^{ref}, is dropped over a distance of $d_{□} \approx 2d_{Debye}$, where d_{Debye} is the Debye length of the Na or Cl ions. From previous measurments[6], we estimate $d_{Debye} \sim 7.5$ nm at our molarity. We then estimate a maximum $F_{□} \approx \Delta V^{ref}/(2d_{Debye}) \approx 30$ mV/nm, very close to the field obtained from PL measurements.

In summary, these optical measurements show that a simple water-based electrolyte used here obviously cannot compete in field strength with the ionic liquid, DEME-TFSI, used in the manuscript. Nevertheless, optical measurements provide another independent experimental confirmation that dual ionic gating produces an out-of-plane electric field and that the strength of this field conforms to the model for field strength used in the manuscript while demonstrating the versatility of devices one can make with our approach.

Supplementary Note 6: Ionic liquid capacitance

The areal capacitance of the IL/2DM interface, *C*, entering Equations 1 and 2 can be measured directly via photoluminescence measurements. We measured PL of 2L MoS_2 covered with IL vs. a single gate, V_g. We tracked the trion peak, the position of which is known to depend on carrier density (Supplementary Figure 12). We then followed the procedure described in the literature[8] to extract the carrier density (*n*) from an applied gate voltage (V_g). From a linear fit to this data, we extract the areal capacitance of the IL/2DM interface to be ~10 μF/cm^2. This value is similar to literature values[5].

Supplementary References

1. Pisoni, R. *et al.* Absence of interlayer tunnel coupling of K-valley electrons in bilayer MoS2. *Phys. Rev. Lett.* **123**, 117702 (2019).

2. Xu, K. & Fullerton-Shirey, S. K. Electric-double-layer-gated transistors based on two-dimensional crystals: recent approaches and advances. *J. Phys. Mater.* **3**, 032001 (2020).

3. Charles Kittel. *Introduction to Solid State Physics*. (John Wiley & Sons, 2004).

4. Wang, Z., Chiu, Y.-H., Honz, K., Mak, K. F. & Shan, J. Electrical Tuning of Interlayer Exciton Gases in WSe2 Bilayers. *Nano Lett.* **18**, 137–143 (2018).

5. Gutiérrez-Lezama, I., Ubrig, N., Ponomarev, E. & Morpurgo, A. F. Ionic gate spectroscopy of 2D semiconductors. *Nat. Rev. Phys.* **3**, 508–519 (2021).

6. Newaz, A. K. M., Markov, D. A., Prasai, D. & Bolotin, K. I. Graphene transistor as a probe for streaming potential. *Nano Lett.* **12**, (2012).

7. Jauregui, L. A. *et al.* Electrical control of interlayer exciton dynamics in atomically thin heterostructures. *Science* **366**, (2019).

8. Chernikov, A. *et al.* Electrical Tuning of Exciton Binding Energies in Monolayer WS2. *Phys. Rev. Lett.* **115**, 126802 (2015).

a) b)

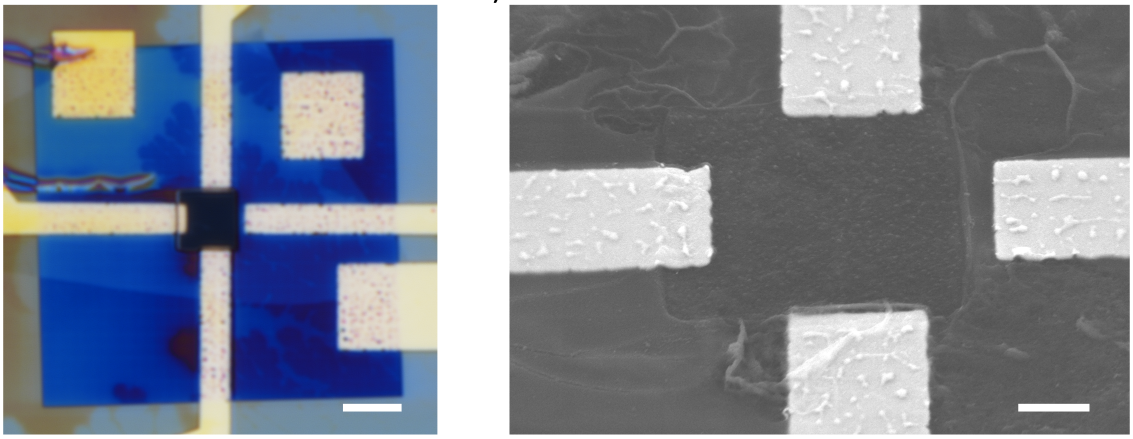

Supplementary Figure 1 | Sample #1, 2L WSe_2, before and after measurement a) The bilayer device before measurement. The black square is the suspended region with side length ~4 µm (length of the scale bar). **b)** Tilted SEM image of the device after measurement (scale bar is 1 µm). The image indicates that the device is not ruptured, and the PMMA visibly covers the metal and supported regions of the flake.

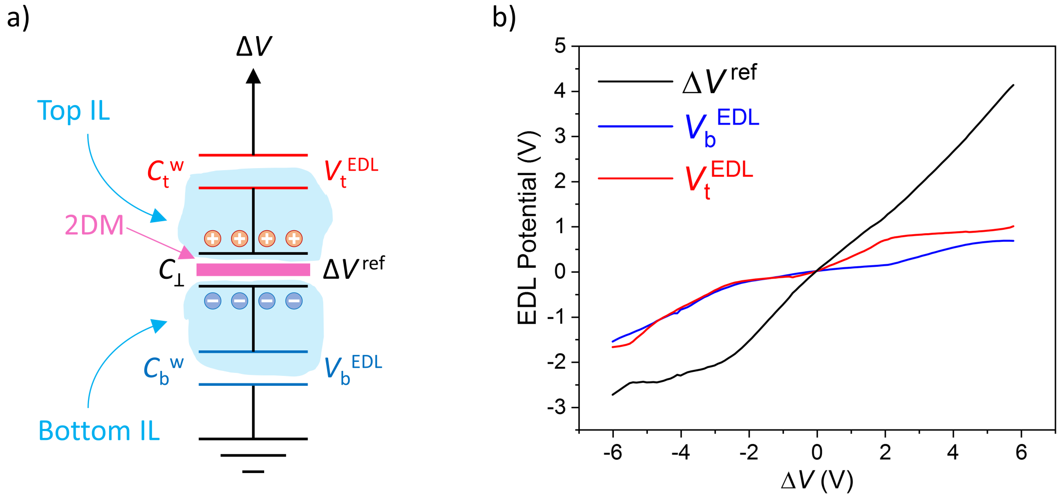

Supplementary Figure 2 | Circuit analysis for reference voltage measurements. a) The circuit for the reference voltage experiment in Fig. 5a of the main text (see Supplementary Figure 5 for complete circuit). The top and bottom gate (working) electrodes have capacitance C_t^w and C_b^w respectively with corresponding voltage drops across their respective EDLs (V_t^{EDL} and V_b^{EDL} respectively), and the capacitance corresponding to the 2D material (2DM) is $C_\perp$ with corresponding voltage drop ΔV^{ref}. **b)** Average voltage drop across the corresponding EDLs as a function of ΔV for each of the three capacitors involved (each curve vertically shifted to be centered about the origin). Note that the two gate EDLs have roughly the same value as a function of ΔV, thereby confirming that $\alpha_b \approx \alpha_t$.

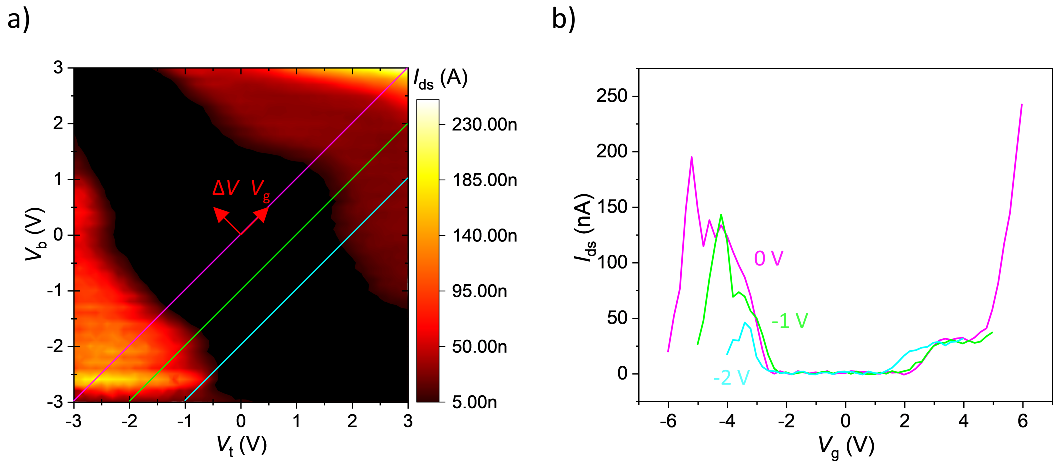

Supplementary Figure 3 | Dual IL-gated monolayer WSe_2. a) Map of I_{ds} vs. (V_b, V_t) for monolayer WSe_2 (sample #5). The rotated coordinate system (V_g, ΔV) is shown at the origin, and diagonal lines indicate constant ΔV of 0 V, 1 V, and 2 V. **b)** Line scans corresponding to the slices of ΔV labeled in the map. Note how the bandgap appears to not change as a perpendicular field is applied. The first few scans of the map in *a)* around $V_b \approx$ -3V are likely hysteretic features, and they appear in the line cuts in *b)* at the lowest V_g values for each curve. These features should be regarded as outliers to the data.

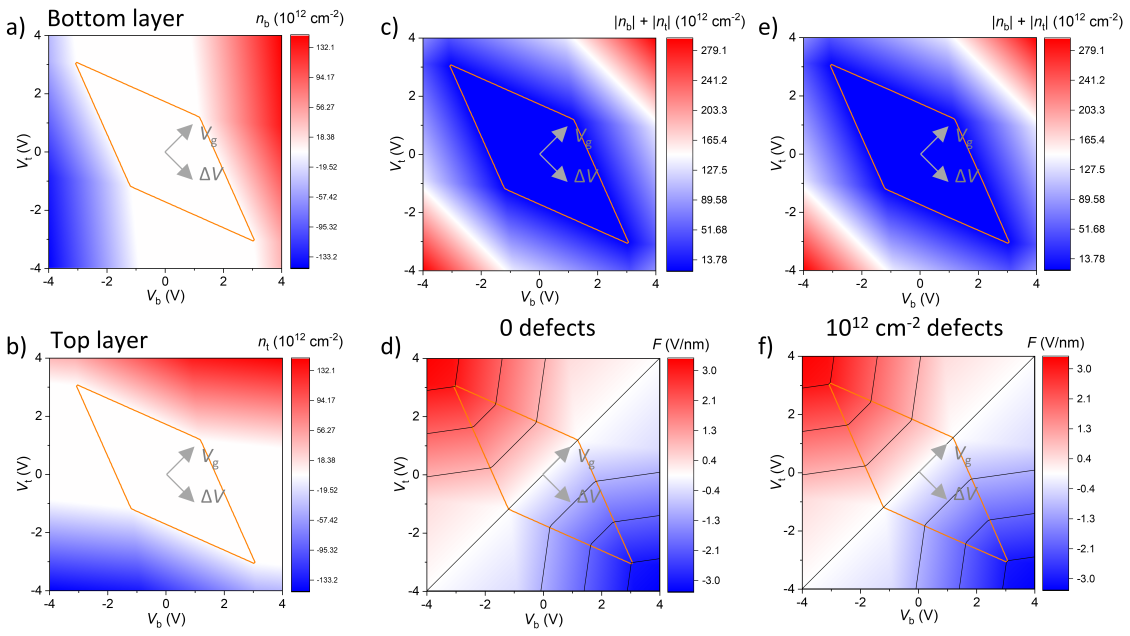

Supplementary Figure 4 | Simulation of field dependent transport in multilayer TMDs. We plot numerically-obtained solution of Equations 1 – 5 for dual-gated bilayer WSe_2. In each plot, the orange diamond outline corresponds to the interlayer bandgap region with zero conductivity. **a), b)** Layer-resolved carrier density for bottom (n_b) and top (n_t) layers respectively. **c)** Total number of carriers in both layers, $|n_b| + |n_t|$, which is proportional to electrical conductivity measured experimentally. The simulation compares to the data of the main text. **d)** Interlayer electric field, F, in the same system, and the contours show field values of 0, ±1, ±2, and ±3 V/nm. Inside the bandgap, the electric field is given by the formula derived in Supplementary Note 1, $F_\perp = (ed_{int})^{-1}(E_{2L}{}^0 - E_{2L})$. Outside of the bandgap, screening effects reduce the field strength. **e), f)** Carrier density and interlayer field for bilayer WSe_2 which has 10^{12} cm^{-2} defects inside the bandgap. Importantly, the presence of this many defects does not affect the transport data or the field strength corresponding to bandgap closing.

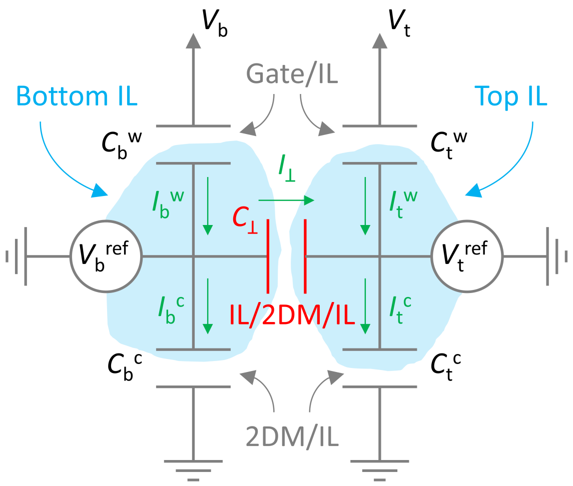

Supplementary Figure 5 | Dual IL-gated FET gating circuit. Dual IL-gating circuit diagram. Each capacitor models either an ion/material or ion/material/ion interface. Each individual IL has its own gate (working) electrode and its own reference voltage. When the 2DM is left floating (circuit in Supplementary Figure 2a), the bottom and top counter electrodes, C_b^c and C_t^c respectively, are left floating, and therefore do not affect the circuit behavior, as in the case of the reference voltage measurements using sample #4 in Fig. 5a of the main text. The leakage current potentially flowing through the device in transport measurements is the sum of the two counter electrode currents, $I_b^c + I_t^c$, which is found from the figure using Kirchhoff's current laws and summing the two measured currents from our sourcemeter: $I_b^w + I_t^w = I_b^c + I_t^c$.

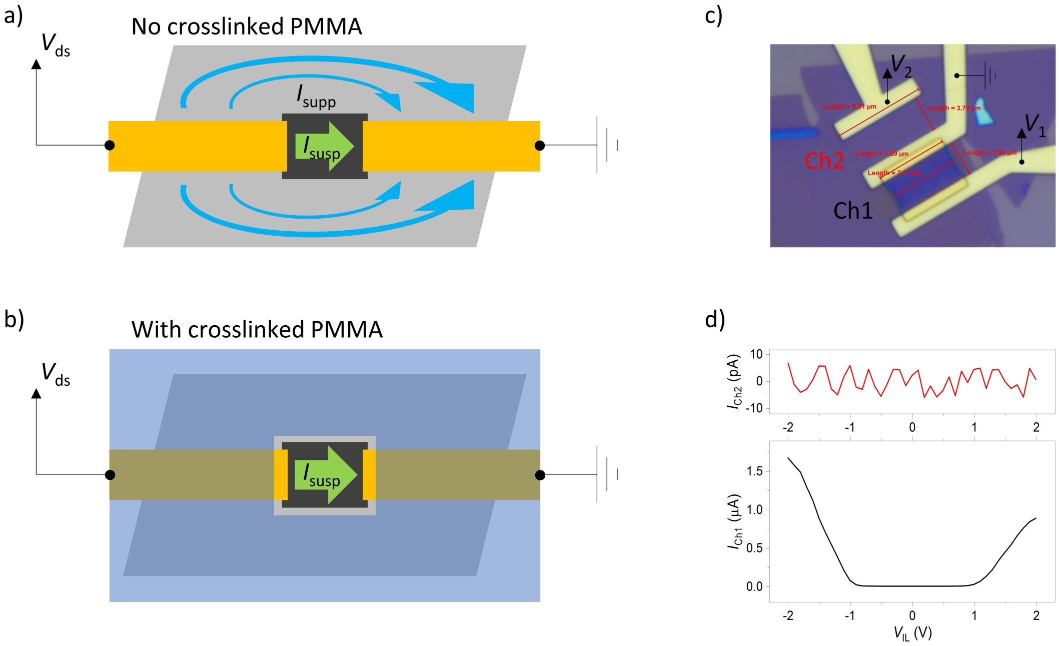

Supplementary Figure 6 | Supported/suspended current and the need for a capping layer. In transport measurements, the total current through our device has contributions from current through supported and suspended regions of the 2DM, given by I_{supp} and I_{susp} respectively. **a)** and **b)** show how supported current is restricted and minimized with the addition of a PMMA capping layer. **c)** 100x image (with dimensions) showing WSe_2 with two channel areas, uncovered (Ch1) and covered (Ch2) with crosslinked PMMA with associated drain-source voltages, V_1 and V_2 respectively. **d)** Electrical transport through both channel regions when $V_1 = V_2$. It is clear from the data that crosslinked PMMA blocks ions from changing the carrier density in covered regions, ensuring that no current will flow in these regions (given that the 2DM is natively charge-neutral). This also shows that our noise floor is ~10 pA, corresponding to the limits of our measurement equipment.

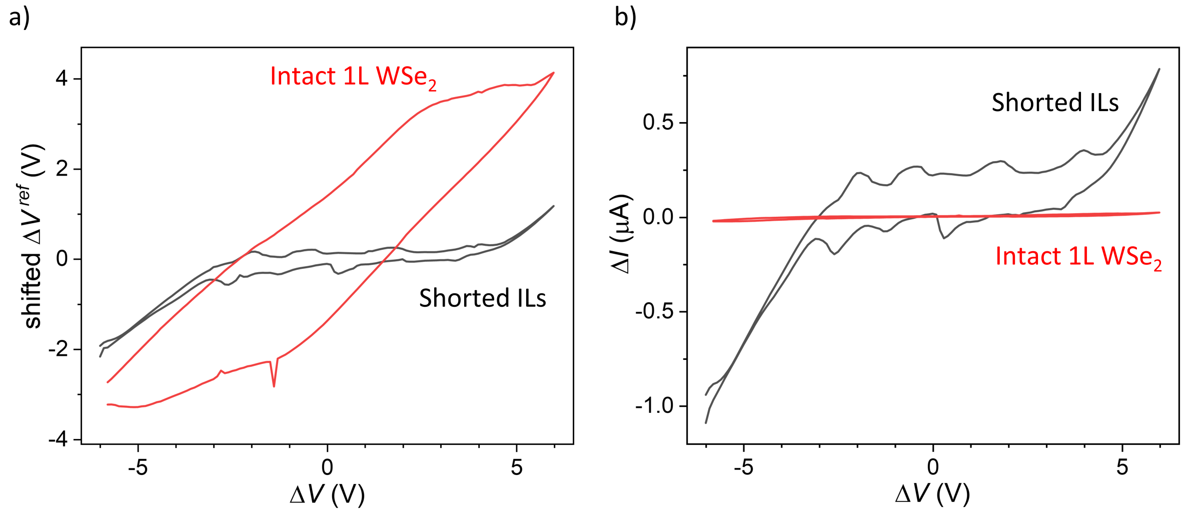

Supplementary Figure 7 | Checking for ruptures in the suspended 2DM. For an intact 1L WSe_2 device (sample #4) and a bare SiN membrane with an uncovered hole in the middle, we show **a)** the (averaged) ΔV^{ref} and **b)** the leakage current (for ΔV). Intact devices have a large reference voltage and a small leakage current, whereas a ruptured device will have a very low reference voltage and large leakage current. Overall, electrochemical measurements allow us to quickly and effectively determine whether a sample is ruptured or not.

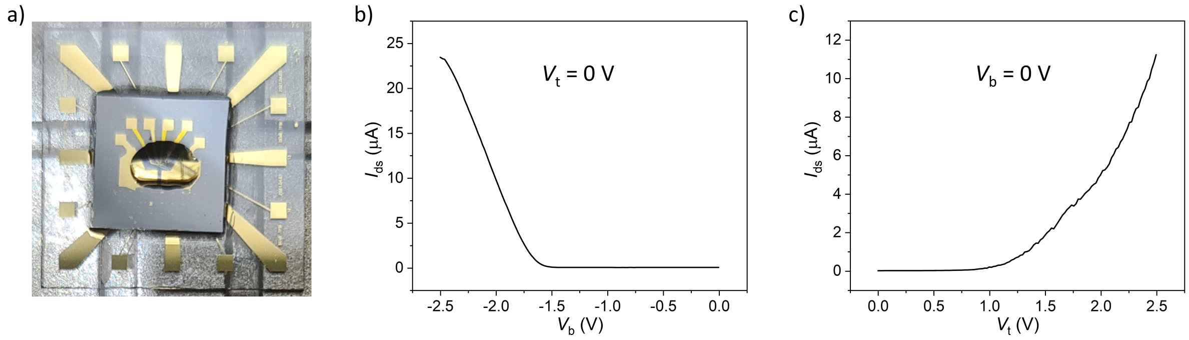

Supplementary Figure 8 | Individual gate operation. For our 4L device, we show **a)** the device assembled in our probe station with visibly-isolated top and bottom ionic liquids, **b)** I_{ds} vs. V_b when V_t = 0 V, and **c)** I_{ds} vs. V_t when V_b = 0 V.

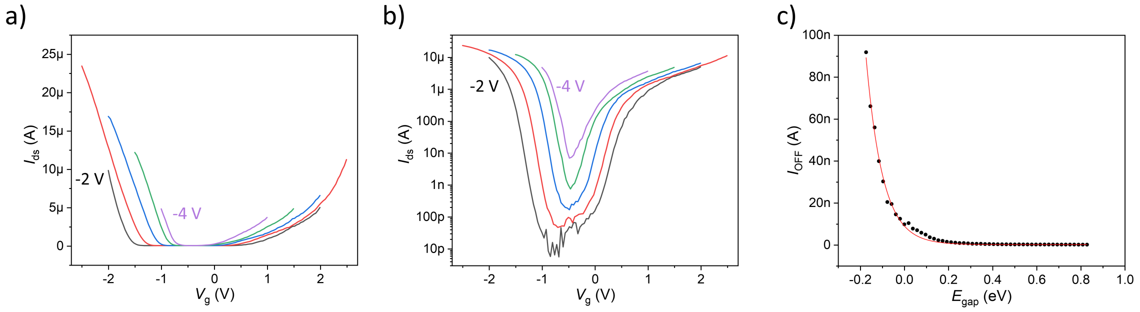

Supplementary Figure 9 | Off-state current near bandgap closing. a) Linear- and **b)** log-scale drain-source current (I_{ds}) vs. V_g of constant ΔV (constant bandgap). These line cuts are from the (V_b, V_t) map from the 4L device in Fig. 4b of the main text. As ΔV increases (E_{gap} decreases), the minimum current in each line cut, I_{OFF}, also increases. **c)** The minimum current, I_{OFF}, at each bandgap, E_{gap}, and exponential fit (red curve).

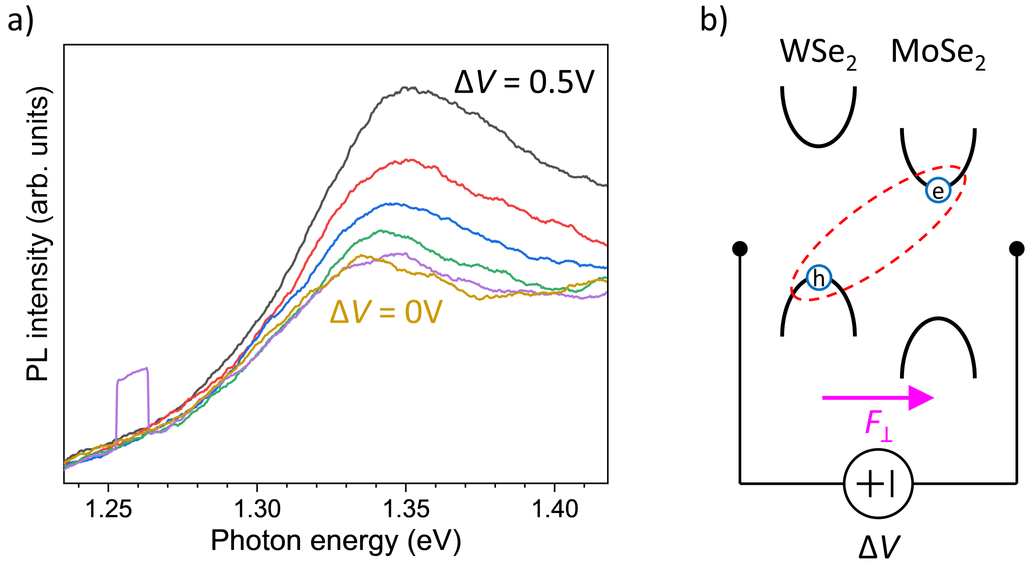

Supplementary Figure 10 | Field-dependent interlayer exciton photoluminescence of a heterobilayer suspended in electrolyte. a) Interlayer exciton photoluminescence of a suspended $MoSe_2/WSe_2$ heterobilayer dual-gated with water/NaCl electrolyte as a function of Δ*V*. The interlayer exciton blueshifts by ~15 meV from 0 V to 0.5 V, corresponding to a field of ~25 mV/nm, closely matching predictions based on the literature. The feature near 1.26 eV is an artifact, a smoothed-out cosmic ray. **b)** Cartoon depicting the orientation of the voltage relative to the TMDC layers. A positive voltage will cause the interlayer bandgap to increase, thereby causing the excitons to blueshift. This measurement demonstrates the versatility of devices one can make with our approach.

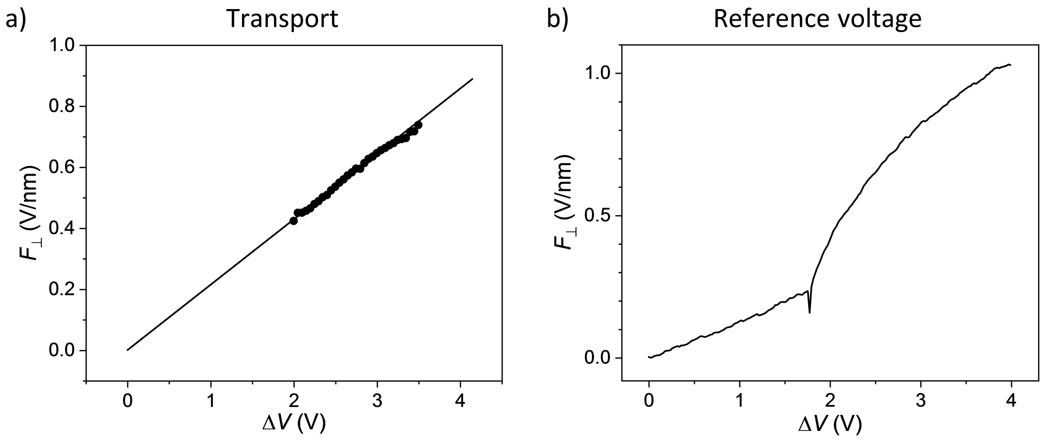

Supplementary Figure 11 | Field from transport vs. field from reference voltage in 4L WSe_2. The field extracted from **a)** transport measurements is very similar to the field extracted from **b)** reference voltage measurements, they both show fields corresponding to bandgap closing (~0.9 V/nm) at $\Delta V \approx 4$ V. This shows that both measurement approaches can accurately measure the field through a (charge-neutral) device.

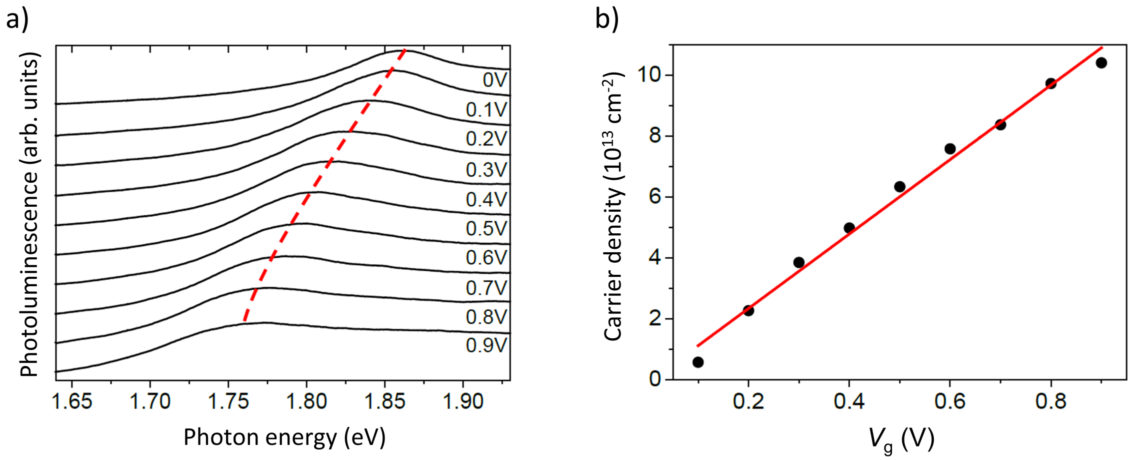

Supplementary Figure 12 | Extracting areal gate capacitance. a) PL spectra of single ionically-gated bilayer MoS_2 vs. gate voltage. The trion peak redshifts with increasing carrier density, allowing us to extract carrier density vs. gate voltage data, shown in **b)**. From the fit to the data (red), we obtain the areal capacitance of the IL/2DM interface, ~10 μF/cm^2.
